# The influence of (central) auditory processing disorder in speech sound disorders^☆^

**DOI:** 10.1016/j.bjorl.2015.01.008

**Published:** 2015-10-20

**Authors:** Tatiane Faria Barrozo, Luciana de Oliveira Pagan-Neves, Nadia Vilela, Renata Mota Mamede Carvallo, Haydée Fiszbein Wertzner

**Affiliations:** a Sciences of Rehabilitation Program, Faculdade de Medicina, Universidade de São Paulo (USP), São Paulo, SP, Brazil; b Speech-Language Pathology and Audiology Program, Department of Physical Therapy, Speech-Language and Audiology and Occupational Therapy, Faculdade de Medicina, Universidade de São Paulo (USP), São Paulo, SP, Brazil

**Keywords:** Articulation disorders, Auditory perception, Speech perception, Evaluation, Transtornos da articulação, Percepção auditiva, Percepção da fala, Avaliação

## Abstract

**Introduction:**

Considering the importance of auditory information for the acquisition and organization of phonological rules, the assessment of (central) auditory processing contributes to both the diagnosis and targeting of speech therapy in children with speech sound disorders.

**Objective:**

To study phonological measures and (central) auditory processing of children with speech sound disorder.

**Methods:**

Clinical and experimental study, with 21 subjects with speech sound disorder aged between 7.0 and 9.11 years, divided into two groups according to their (central) auditory processing disorder. The assessment comprised tests of phonology, speech inconsistency, and metalinguistic abilities.

**Results:**

The group with (central) auditory processing disorder demonstrated greater severity of speech sound disorder. The cutoff value obtained for the process density index was the one that best characterized the occurrence of phonological processes for children above 7 years of age.

**Conclusion:**

The comparison among the tests evaluated between the two groups showed differences in some phonological and metalinguistic abilities. Children with an index value above 0.54 demonstrated strong tendencies towards presenting a (central) auditory processing disorder, and this measure was effective to indicate the need for evaluation in children with speech sound disorder.

## Resumo

**Introdução:**

Considerando a importância das informações recebidas auditivamente para a aquisição e organização das regras fonológicas, a avaliação do processamento auditivo (central) traz contribuições significativas para o diagnóstico e direcionamento da intervenção fonoaudiologia das crianças com transtorno fonológico.

**Objetivo:**

Estudar as medidas fonológicas e o processamento auditivo (central) de crianças com transtorno fonológico.

**Método:**

Estudo clínico e experimental com 21 sujeitos com transtorno fonológico, entre 7,0 e 9,11 anos, separados em dois grupos: com e sem transtorno do processamento auditivo (central). Foram avaliadas as provas de fonologia, inconsistência de fala e habilidades metalinguísticas.

**Resultados:**

O grupo com transtorno do processamento auditivo (central) apresentou maior gravidade do transtorno fonológico. O valor de corte obtido para o process density index foi o que melhor caracterizou a ocorrência dos processos fonológicos para crianças acima de sete anos.

**Conclusão:**

A comparação do desempenho das provas avaliadas nos dois grupos mostrou diferenças quanto a alguns aspectos fonológicos e metalinguísticas. As crianças com valor do índice acima de 0,54 demonstraram uma forte tendência a apresentar alteração no processamento auditivo (central), sendo que esta medida foi efetiva para indicar a necessidade de avaliação de crianças com transtorno fonológico.

## Introduction

Several aspects have been explored in the studies of children with speech sound disorder (SSD), which is a speech disorder characterized by an inadequate use of phonological rules of language (DSM-IV F80.0 – 315.39). The dynamic models that attempt to explain the development of speech production indicate an interaction between auditory perception, sound production, and sound representation.^1^, ^2^ Thus, a detailed observation of the performance of children with SSD regarding central auditory processing skills can contribute a great deal to the understanding of speech and language manifestations. The central question of this study was to investigate whether children with SSD who were diagnosed late (between 7 and 9 years, 11 months) also have (central) auditory processing disorders.

An impairment in the phonological system is the main feature of SSD and may stem from specific difficulties related to cognitive-linguistic processing (organization of phonological rules), auditory processing, and/or speech production. The interrelationship among these three processings has been the subject of several studies^3^, ^4^, ^5^ that sought to improve the understanding of the manifestations observed in children with SSD.

Regardless of the SSD classification system used, the literature points to the existence of subtypes,^6^, ^7^ demonstrating a variety of difficulties that may exhibit different manifestations and varying degrees of expression. Such manifestations can be identified by various tests complementary to phonological tests; for instance, speech inconsistency, metalinguistic skills, and those involving auditory organization, assessed in (central) auditory processing (CAP) tests.

The classification of SSD severity is a complex task, because the clinician must consider the phonological changes, speech intelligibility, and age of the child, among other factors. Some severity index classifications can be found in the literature, such as the Percentage of Consonants Correct (PCC),^8^ its revised version, the PCC-R,^9^ and the Process Density Index (PDI).^10^

Both PCC and PCC-R are intended to indicate the percentage of correct consonants in a conventional speech sample. The main difference between these tools is the fact that PCC considers substitutions, omissions, and distortions as speech errors, while PCC-R considers only consonant substitutions and omissions as errors.

PDI verifies the occurrence of phonological processes, and is distinct from PCC and PCC-R, inasmuch as these two latter indexes account for the correct consonants of speech samples. PDI is inversely proportional to PCC and to PCC-R, in that the lower the value of PCC (or of PCC-R), the higher the value of PDI; that is, the lower the percentage of correct consonants employed in speech, the higher the frequency of use of phonological processes.^11^

PCC-R and PDI have been applied on Brazilian Portuguese (BP)-speaking children with SSD in the same region of the country where the children of this study live. Studies show that this is an efficient index for classifying SSD severity.^12^, ^13^, ^14^

Intelligible speech depends on efficient phonological programming, which reflects the individual's ability to select the target phoneme and organize the sounds in the correct sequence.^15^ Difficulty in phonological programming can be evaluated by the speech inconsistency test,^12^, ^15^ which indicates a possible deficit in cognitive-linguistic processing that interferes with the internalization of phonological rules of the language the child is exposed to.

An important feature of school-age children with SSD is that they usually show difficulties related to phonological processing; therefore, analyzing the awareness of smaller units that make up speech is an important step in evaluating these children. Several metalinguistic skills are assessed in school-age children, including rhyme and alliteration. The Phonological Sensitivity Test (PST)^16^ checks the phonological coding strategies of the child, through an evaluation of metalinguistic skills of rhyme and alliteration (equal and different). This test has two versions: auditory (with auditory support) and visual (with both auditory and visual support), which was designed to verify if children with SSD benefit from visual support.

To further complement the identification of difficulties faced by children with SSD and considering the interaction of auditory information received – in association with the acquisition and organization of phonological rules in this population – the evaluation of CAP brings significant contributions to the diagnosis of SSD and targeting for phonological intervention. This contribution occurs because (central) auditory processing disorder (CAPD), defined as a difficulty in processing sound information, may result in a difficulty in language development and in learning.^17^

Although auditory difficulties are the primary complaints of children with CAPD, one study^18^ observed that other impairments can be identified, such as those related to language, to reading and writing, and also to learning difficulties.

Importantly, there are few studies that correlate SSD with CAP. This may be justified by the fact that SSD diagnosis is most often established in children aged 5–7 years, and also because the application of the CAP test is performed only after 7 years of age, due to necessity for the maturation of the structures involved.

Studies^19^, ^20^ in children with other language impairments – for instance, specific language impairment and dyslexia – showed the importance of CAP assessment for complementing diagnosis, as children with these disorders may exhibit CAPD with an impairment in skills involving discrimination of speech sounds, and that may result in impaired and/or less stable neural representations of the sound, perhaps interfering with perception and speech production.

Several tests that assess auditory skills and that are already adapted to the Brazilian Portuguese language are available. Some of them have more use in the evaluation of CAP in children with SSD, by assessing specific speech understanding skills, without their results suffering interference from the phonological change presented by the child. Among these tests, the following are notable: Picture Identification with White Noise, which evaluates auditory closure ability; Dichotic Digit Test, which evaluates figure-ground ability; and Frequency Pattern and Duration Pattern Tests, which assess the hearing abilities of temporal ordering and interhemispheric transfer.

Faced with the diversity of correlated causes and of phonological manifestations found in children with SSD, it is critical to obtain more detailed descriptions on aspects that complement the phonological evaluation in these children.

The aim of this study was to investigate phonological measures and (central) auditory processing of children with speech sound disorder.

## Methods

The study was approved by the Research Ethics Committee of the university where the study was conducted (No. 201/11). An informed consent was signed by the parent or guardian of each child.

Twenty-one subjects (both genders) diagnosed with SSD, with ages between 7.0 and 9.11 years, were included in this study. The diagnosis was established in a specialized laboratory linked to the university where the study was conducted.

According to CAP evaluation results, the subjects were allocated to the control group (CG), with 10 subjects without CAPD, or to the study group (SG), with 11 subjects with CAPD. As inclusion criteria, the child needed to have speech errors in the phonological test^21^ and an age appropriate performance on vocabulary, fluency, and pragmatic evaluations of the ABFW Child Language Test in phonology, vocabulary, fluency, and pragmatic areas^22^; needed to have completed the (central) auditory processing (CAP) examination^23^; needed to have his/her hearing thresholds within the normal range; and could not have undergone speech therapy. In addition, the Speech Inconsistency Test (SIT)^2^ and the PST were applied.^16^ All participants were Brazilian Portuguese-speaking subjects.

The phonology evaluation of the ABFW^21^ consists of the picture naming task (N) that includes 34 figures with 90 correct consonants, and the imitation of words task (I) with 39 words totaling 107 correct consonants. The two phonology tests were transcribed twice by phonology researchers. The agreement between the transcripts was 90%. From the phonological tests, PCC,^8^ PCC-R,^9^ and PDI^10^ severity indexes, the number of different types of phonological processes, as well as the occurrence of each process, were calculated. The following phonological processes were analyzed: syllable reduction (SR); consonant harmony (CH); stopping (S); velar backing (VB); palatal backing (PB); velar fronting (VF); palatal fronting (PF); liquid simplification (LS); cluster reduction (CR); final consonant deletion (FCD); stop voicing (SV); fricative voicing (FV); stop devoicing (SD); and fricative devoicing (FD).

SIT^12^ consists of 25 pictures named three times in different sequences, interspersed by distracting activities. The three namings of each word were analyzed and classified, and the subject was considered as consistent when he/she named the picture equally in three different times, and as inconsistent when at least one of the namings was performed differently. The speech inconsistency index^12^ represents the percentage of inconsistent words in the test and was analyzed according to the criteria established by the authors, determining who is consistent or inconsistent, considering the established cut-off values according to gender and age. Subjects were considered inconsistent when they achieved inconsistency index rates below the cutoff values. For girls aged 5.0–7.6 years, the cutoff value is ≥21.5%; for the age group above 7.6 years, it is ≥14.5%. For boys aged 5.0–7.6 years, the cutoff value is ≥31.9%; for the age group above 7.6 years, it is ≥17.6%.

For the evaluation of metalinguistic skills (rhyme and alliteration, equal and different), the PST^16^ in its auditory and visual versions was applied. This is a test divided into four parts that verify the performance in rhyme (equal and different) and alliteration (equal and different) skills. Each test consists of 15 items; the first three items are used to explain the test and the following 12 items, for application and analysis of responses. In the alliteration parts, the subject is requested to say which of the three words begins equally or differently from the target word; in the rhyme test, the subject is asked to say which of the three words ends equally or differently from the target word. The maximum value for correct answers for each test is 12.

The present study used both the Auditory Version (PST-A), wherein the subject has only auditory support when answering, and the Visual Version (PST-V), in which the subject has auditory support associated to visual support (pictures). The application of the test was divided into four sessions, to prevent interference of the subject's fatigue with the results, which were analyzed according to the criteria established in the literature.^16^

The Picture Identification Tests with White Noise,^24^ Dichotic Digit Test,^25^ Frequency Pattern Test,^26^ and Duration Pattern Test^26^ were employed for the assessment of CAP. The criterion for identifying APD in the tested subjects was an observed change in at least two of the four administered tests.^27^

The evaluation of CAP was carried out in a specialized laboratory from the same university where the study was conducted. To perform this test, we used a GSI-61 Grason-Stadler audiometer with frequency range from 125 to 12,000 Hz, and an intensity variation of 10–110 dB HL for pure tone in the frequencies of 125 Hz and 12,000 Hz, and of −10 to 120 dB HL for the frequencies of 500, 750, 1000, 2000, 3000, 4000, 5000, and 6000 Hz. The calibration was carried out according to ANSI S3 6-1989; ANSI S3 43; IEC 645-1 (1992); IEC 645-2 (1993) and ISSO 389; UL 544, and was conducted in a soundproof booth (Siemens) calibrated according to ANSI S3 1-1991 standards.

### Statistical method

The following statistical tests were used: Fisher's exact test, Student's *t*-test, and the Mann–Whitney test. As to hypothesis tests, the significance level was set at 0.05. The analysis was performed using Minitab (version 16) and SPSS (version 18) statistical programs.

## Results

In the analysis performed by gender in both groups, the results showed that although there was no difference between percentage distribution for this variable (*p* > 0.999, Fisher's exact test), it was noted that most of the subjects were male, both in the CG (7) and the SG (8).

Regarding the subjects’ age, the values were CG (8.5) and SG (7.10); no significant differences between means of the groups were observed (*p* = 0.131, Student's *t*-test).

As to the number of different types of phonological processes in phonological tests, the results showed that SG participants used a mean of four types of phonological processes in each of the tasks. Conversely, CG participants used a mean of three types of phonological processes. Although the SG had a mean higher number of phonological processes regardless of phonological test, this difference was not significant with respect to imitation of words (*p* = 0.458) or to picture naming (*p* = 0.538).

The descriptive values for the percentage of occurrence of each phonological process in both phonological tasks (Table 1) show that the phonological processes with the highest mean percentage of occurrence were LS, CR, FCD, SD, and FD. Depending on these results, the distributions of these processes were compared between the two groups (CG and SG; Table 2), and a difference was found only for CR in the word imitation test, indicating a higher occurrence of this process in SG.

**Table 1:** Descriptive values for percentage of occurrence of phonological processes in phonology tasks in the control group (CG) and study group (SG).

Imitation of words task					Picture naming task				
Phonological process	Group	*n*	Mean	Standard deviation	Phonological process	Group	*n*	Mean	Standard deviation
SR	No occurrence				SR	CG	10	0.2	0.7
						SG	11	0.2	0.7
CH	CG	10	0.2	0.6	CH	CG	10	0.2	0.7
	SG	11	0	0		SG	11	0	0
S	CG	10	0	0	S	CG	10	0.4	1.4
	SG	11	0.4	1.4		SG	11	1.2	2
VB	CG	10	0	0	VB	CG	10	0	0
	SG	11	5.6	18.5		SG	11	6.1	20.1
PB	No occurrence				PB	CG	10	4.6	11.6
						SG	11	4.1	13.7
VF	CG	10	0.2	0.6	VF	CG	10	0	0
	SG	11	27.3	46.7		SG	11	26.3	43.1
PF	CG	10	16.7	31.4	PF	CG	10	20	26.7
	SG	11	3	10		SG	11	0	0
LS	CG	10	16.3	21.3	LS	CG	10	22.7	26.8
	SG	11	15.9	14.9		SG	11	14.9	19.2
CR	CG	10	29.2	27	CR	CG	10	30	29.6
	SG	11	65.9	41.1		SG	11	55.7	40.8
FCD	CG	10	10	13.5	FCD	CG	10	10	21.6
	SG	11	20.8	19.5		SG	11	27.3	27.2
SV	CG	10	0.3	1.1	SV	No occurrence			
	SG	11	0.6	1.4					
SD	CG	10	22.9	37.1	SD	CG	10	22.1	39.8
	SG	11	18.2	34		SG	11	20.1	38
FD	CG	10	24.4	36.2	FD	CG	10	24.4	38.4
	SG	11	23.2	33.5		SG	11	28.3	44

*n*, number of subjects; SR, syllable reduction; CH, consonant harmony; S, stopping; VB, velar backing; PB, palatal backing; VF, velar fronting; PF, palatal fronting; LS, liquid simplification; CR, cluster reduction; FCD, final consonant deletion; SV, stop voicing; SD, stop devoicing; FD, fricative devoicing.

**Table 2:** *p*-Values obtained in the comparison of distribution of LS, CR, FCD, SD, and FD processes between Control Group and Study Group.

Phonological process	Phonology task	
	Imitation of words	Picture naming
LS	0.88	0.555
CR	0.041^a^	0.079
FCD	0.231	0.123
SD	0.852	0.938
FD	0.879	0.754

LS, liquid simplification; CR, cluster reduction; FCD, final consonant deletion; SD, stop devoicing; FD, fricative devoicing.

a Significant difference.

**Figure 1:**
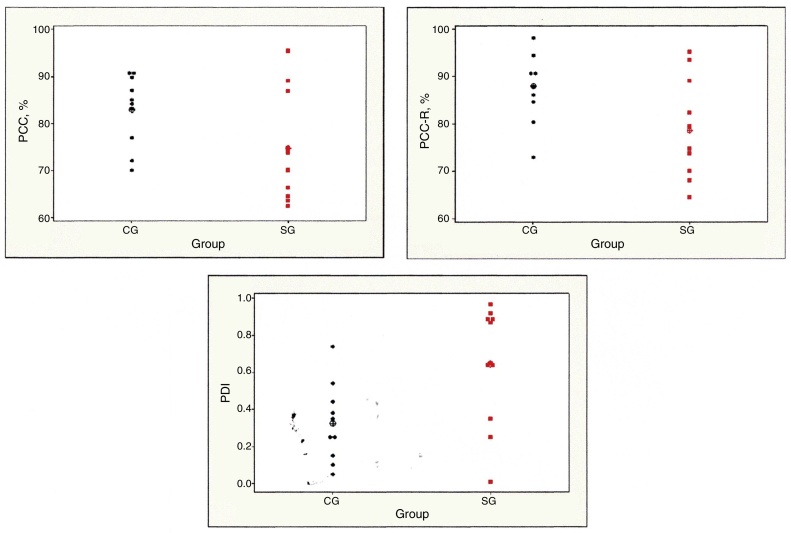
Individual and mean values of Percentage of Consonants Correct (PCC), Percentage of Consonants Correct-Revised (PCC-R), and Process Density Index (PDI) in the control group (CG) and study group (SG).

The descriptive values of PCC, PCC-R, and PDI in each group (Table 3) showed that, for all severity indexes, there was a difference in the comparison between groups. It can be observed that the mean values of the PCC and PCC-R were lower in SG, and those of the PDI were higher in this group (Fig. 1).

**Table 3:** Descriptive statistics for Percentage of Consonants Correct (PCC), Percentage of Consonants Correct-Revised (PCC-R), and Process Density Index (PDI) in the control group (CG) and study group (SG).

Group	*n*	Mean	Standard deviation	Minimum	Median	Maximum	*p*-Value
*PCC*							
CG	10	82.9	7.5	70.1	84.6	90.7	0.031^a^
SG	11	74.7	11.1	62.6	73.8	95.3	

*PCC-R*							
CG	10	88	7.5	72.9	89.3	98.1	0.014^a^
SG	11	78.6	10.3	64.5	74.8	95.3	

*PDI*							
CG	10	0.33	0.21	0.1	0.3	0.7	0.007^a^
SG	11	0.64	0.32	0	0.65	1	

*n*, number of subjects.

Statistics: Student's *t*-test.

a Significant difference.

**Figure 2:**
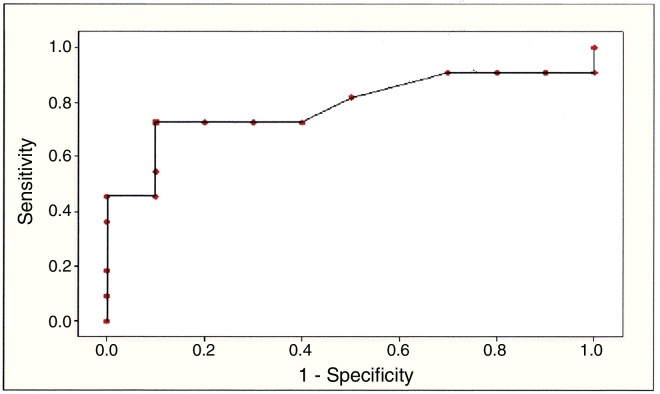
Receiver operating characteristic (ROC) curve for PDI.

For the PDI index, a receiver operating characteristic (ROC) curve was drawn (Fig. 2), indicating the nearest point of the upper left corner corresponded to the higher sensitivity (0.73) and specificity (0.90) values. A cutoff value of 0.54 was associated with this point. The area under the curve (AUC) of 0.79 confirmed the discriminatory power of the PDI. Thus, subjects with PDI ≥ 0.54 are most likely to belong to SG, i.e., to present an AP change.

The results of SIT classification suggested that, in both groups, the number of consistent children was higher: six in the CG and nine in the SG. However, no difference between the percentages of occurrence of inconsistent subjects in both groups (*p* = 0.268, Fisher's exact test) was found.

Distributions of frequencies and percentages of PST-A and PST-V in CG and SG (Table 4) showed no difference between percentages of impaired results in the four tests in both versions, both in the CG (*p* = 0.504) and the SG (*p* = 0.772, analysis of variance); however the percentage of impaired results was greater in the SG (*p* < 0.001, Fisher's exact test) in all PST-A tasks. In PST-V, the percentages of impaired results were higher in the SG (*p* < 0.001, Fisher's exact test), and this result was independent of the task (*p* = 0.196, analysis of variance).

**Table 4:** Frequency distributions and percentages of Phonological Sensitivity Test-Auditory (PST-A) and Phonological Sensitivity Test-Visual (PST-V) in the control group (CG) and study group (SG).

	CG				SG			
	Impaired		Normal		Impaired		Normal	
	*n*	%	*n*	%	*n*	%	*n*	%
**PST-A**								
*Initial sound*								
Equal	1	10	9	90	7	63.6	4	36.4
Different	0	0	10	100	7	63.6	4	36.4

*Final sound*								
Equal	1	10	9	90	9	81.8	2	18.2
Different	1	10	9	90	8	72.7	3	27.3

**PST-V**								
*Initial sound*								
Equal	0	0	10	100	6	54.6	5	45.4
Different	0	0	10	100	3	27.3	8	72.7

*Final sound*								
Equal	2	20	8	80	7	63.6	4	36.4
Different	0	0	10	100	8	72.7	3	27.3

*n*, number of subjects.

Comparing PST-A *vs.* PST-V, a *p*-value of 0.095 was found; the lack of statistical power can be attributed to the small sample size in both groups.

## Discussion

The manifestations of SSD are heterogeneous, which makes their classification difficult.^4^ Thus, the present study described the performance of children diagnosed with SSD in various phonological (number of different types of phonological processes, SSD severity, and speech inconsistency) and metalinguistic (PST-V and PST-A) skills, depending of the presence/absence of impairment in CAP.

The description of both groups, CG and SG, indicated no difference regarding the subjects’ age; a male gender predominance was noted in both groups.

Among the phonological measures examined, the number of different types of phonological processes has shown no evidence of difference between CG and SG in both phonological tasks. Nevertheless, SG presented higher means for this variable, both in imitation of words as well as in picture naming tasks.

As for phonological process types with higher incidence, in general, the same findings already noted in previous studies with Brazilian Portuguese-speaking children were observed: SD, FD, LS, and CR.^13^ Considering that the present study compared the performance of children with SSD with and without CAPD, we found that CR was observed most frequently among these phonological processes, both in the CG and the SG. In addition, CR was the only phonological process that showed difference only in imitation of words test between the two groups, with greater occurrence in the SG, suggesting that a CAPD can hinder the phonological organization of complex structures.

Nonetheless, in the analysis of the three severity indices (PCC, PCC-R, and PDI), SG subjects presented higher SSD severity compared to subjects without CAPD, i.e., SG subjects had fewer correct consonants and higher incidence of phonological processes. In a study conducted among Brazilian Portuguese-speaking children with SSD^28^ and without CAPD, higher and more homogeneous values were found in the PCC-R index, suggesting that children with SSD and CAPD show higher SSD severity, i.e., greater phonological difficulty.

Evidence arising from the comparison of severity measures and phonological processes in children with SSD with and without CAPD is critical because, in general, very little is discussed about CAP in these children. Whereas studies using dynamic models related to the development of speech are advancing, the interaction between auditory perception and the production and phonological organization of speech sounds are becoming more appreciated.^1^, ^2^ Accordingly, a verification of the relationship among phonological and CAP measurements in children over 7 years of age with SSD shows that searching for this relationship is not only appropriate but also necessary.

According to the literature,^20^ close relationships are observed between speech impairment and CAPD, since CAP hinders the formation of phonemic representation in the brain, thus interfering with the learning of the rules of phonology, syntax, and semantics. The fact that SG subjects with CAPD employed a greater number of different types of phonological processes indicates greater difficulty in phonological representation, perhaps due to the difficulty that these subjects seem to have in retrieving the phonological representations through auditory feedback during speech production.^1^

Among the indexes that characterize SSD severity, PDI was the one that best characterized the occurrence of all phonological processes in speech. Due to this finding, a ROC curve was constructed for PDI, in order to explore in greater detail the occurrence of phonological processes due to CAPD. The cutoff value identified suggests that children with SSD aged 7 years and presenting PDI ≥ 0.54 may present CAPD. This cutoff value provides evidence that PDI, applied to speech samples at the time of the SSD diagnosis for children over 7 years of age, is effective to identify children in need of referral, on a priority basis, for evaluating CAP. By evaluating CAP, additional information can be obtained, greatly assisting the planning and execution of the treatment of each child.

The last phonological measure evaluated in the study – the speech inconsistency classification – was analyzed in order to determine whether children with inconsistent SSD had CAPD. Our study revealed no difference between groups and that, independently of the group, the number of consistent subjects was greater than the number of inconsistent ones. This result indicates that, for this sample of subjects, phonological programming did not suffer interference of CAPD. Studies^12^, ^29^ that have already applied the speech inconsistency classification indicate that most children with SSD are consistent, i.e., they do not experience difficulty in phonological programming.

Regarding tests evaluating the metalinguistic skills of rhyme and alliteration, the study showed that the level of impaired results was higher in the SG in the four subtests and in both versions of the PST. This finding suggests a relationship between the skills involved in CAP with those of PST, i.e., if the child shows a CAPD, it is more likely that difficulties will occur in the metalinguistic skills of rhyme and alliteration. This can be explained by the greater difficulty of perception and of auditory organization presented by SG subjects, which interferes with the ability to answer each item correctly, as in the case of the PST, the child needs to retain the stimulus in its working memory and recognize if the initial or final sound is equal or different. This finding suggests an interrelationship of the processings involved in speech.^1^, ^3^ Concerning PST, the present study compared the two versions of the test (visual and auditory), and found no difference between these versions in the two groups. Thus, the visual support provided by the pictures presented in PST-V did not help the performance of children with SSD and CAPD.

This study was designed to be able to ascertain if the phonological and metalinguistic manifestations of children with SSD differed according to the presence of a CAPD. The study of such relationship contribute to a more accurate diagnosis of SSD and to more effective interventions for children with this disorder. The analyses performed for phonological and metalinguistic measures indicated that children with SSD and CAPD have a more severe condition, present greater use of the CR phonological process, have more difficulty in rhyme and alliteration skills, and also did not benefit from visual cues for these skills.

An important finding of this study was the cutoff point established for the PDI, which effectively differentiated children with SSD and CAPD from those with SSD without CAPD. Therefore, PDI, which is an index that measures the occurrence of phonological processes in a speech sample, can be applied to the evaluation of SSD diagnosis, suggesting the need to evaluate the CAP in children with SSD aged over 7 years.

The principal limitation of this study was the relatively small number of participants, since the age required for assessing CAP is over 7 years, and the diagnosis of SSD is most often made before that age. That is the reason why the number of children who met the inclusion criterion of age was reduced.

This study is innovative because it demonstrated that children with SSD with a PDI value > 0.54 exhibit a strong tendency to present CAPD.

## Conclusion

A comparison of performances of children with SSD with and without CAPD showed evidence of differences among them on some phonological and metalinguistic skills. Children with SSD and CAPD showed higher occurrence of the phonological process of CR, greater difficulty in rhyme and alliteration tests and, in addition, were not benefited by the pictures provided at the PST-V. They had lower PCC-R and higher PDI values. In addition, children with SSD with a PDI value above 0.54 demonstrated a strong tendency to have CAPD, and this measure was effective to identify children with SSD in need of a CAP evaluation.

## Funding

This study was funded by CAPES – Institutional Quota (Social Demand) – Universidade de São Paulo.

## Conflicts of interest

The authors declare no conflicts of interest.
